# Heterologous production of the D-cycloserine intermediate O-acetyl-L-serine in a human type II pulmonary cell model

**DOI:** 10.1038/s41598-023-35632-4

**Published:** 2023-05-26

**Authors:** Laurel Robbins, Ariane Balaram, Stefanie Dejneka, Matthew McMahon, Zarina Najibi, Peter Pawlowicz, William H. Conrad

**Affiliations:** Department of Chemistry and Biochemistry and Molecular Biology Program, Lake Forest College, Lake Forest, USA

**Keywords:** Enzymes, Drug delivery, Tuberculosis, Drug development, Genomic engineering, Metabolic engineering, Molecular engineering, Protein engineering, Synthetic biology, Applied microbiology, Biochemistry, Drug discovery, Microbiology

## Abstract

Tuberculosis (TB) is the second leading cause of death by a single infectious disease behind COVID-19. Despite a century of effort, the current TB vaccine does not effectively prevent pulmonary TB, promote herd immunity, or prevent transmission. Therefore, alternative approaches are needed. We seek to develop a cell therapy that produces an effective antibiotic in response to TB infection. D-cycloserine (D-CS) is a second-line antibiotic for TB that inhibits bacterial cell wall synthesis. We have determined D-CS to be the optimal candidate for anti-TB cell therapy due to its effectiveness against TB, relatively short biosynthetic pathway, and its low-resistance incidence. The first committed step towards D-CS synthesis is catalyzed by the L-serine-O-acetyltransferase (DcsE) which converts L-serine and acetyl-CoA to O-acetyl-L-serine (L-OAS). To test if the D-CS pathway could be an effective prophylaxis for TB, we endeavored to express functional DcsE in A549 cells as a human pulmonary model. We observed DcsE-FLAG-GFP expression using fluorescence microscopy. DcsE purified from A549 cells catalyzed the synthesis of L-OAS as observed by HPLC–MS. Therefore, human cells synthesize functional DcsE capable of converting L-serine and acetyl-CoA to L-OAS demonstrating the first step towards D-CS production in human cells.

## Introduction

Tuberculosis (TB) is a primarily respiratory infectious disease caused by the bacterium *Mycobacterium tuberculosis* (Mtb)^1^.TB remains one of the leading causes of death by a communicable disease worldwide, infecting 10 million people and killing 1.5 million people in 2021^2^. The current Bacillus Calmette–Guérin (BCG) vaccine for TB has variable protection rates for pulmonary TB that do not last into adulthood and can lead to adverse effects (for review see ref.^3^). Other vaccine attempts, such as the MVA85A vaccine, were developed to supplement BCG but failed in phase IIIb trials, therefore, TB prophylaxis remains elusive^4^.

Developing an effective TB prophylaxis presents an important opportunity for alternative approaches. The successful history of chemoprophylaxis and emerging gene therapies have laid the foundation for the possibility of developing a genetic prophylaxis that produces an antibiotic in human cells (genetic chemoprophylaxis). Isoniazid has been used as a chemoprophylaxis that decreases the odds of developing TB by 40% over 2 or more years^5^. Gene therapies have recently been discovered efficacious against cancer. Chimeric Antigen Receptor T-Cell (CAR-T) therapy genetically modifies T-cells to include a chimeric antigen receptor. This helps T-cells target cancer cells, specifically acute lymphoblastic leukemia and large B-cell lymphomas^6^. In a clinical trial enrolling children with B acute lymphoblastic leukemia for CAR T-22 treatments, 17 out of 20 participants achieved at least one year remission^7^. After CAR-T therapy demonstrated that it is possible to genetically modify cells to target cancer cells, numerous alternative delivery methods and cell based therapies have been developed^8^. Because of the effectiveness of chemoprophylaxis and the emergence of gene therapy, we seek to explore the feasibility of a genetic chemoprophylaxis that produces an antibiotic to prevent TB.

**Figure 1:**
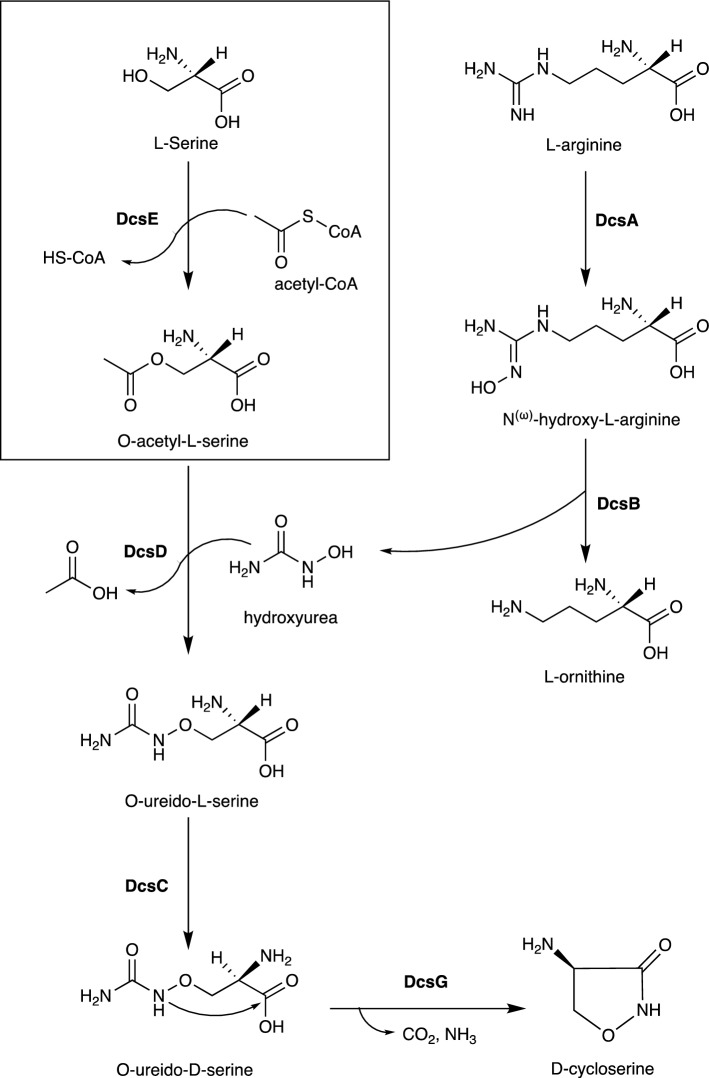
Biosynthetic pathway for D-CS^9,14^. Boxed reaction catalyzed by DcsE. D-cycloserine can be produced biosynthetically using six enzymes, DcsA-G, (in bold) and biologically available reagents: L-serine, L-arginine, and acetyl-CoA^12^.

After evaluating all first- and second-line TB antibiotics, we selected the D-cycloserine (D-CS) biosynthetic pathway as an optimal candidate for genetic chemoprophylaxis. In this study, we focused on the first enzyme in the biosynthetic pathway, L-serine-O-acetyltransferase (DcsE), which produces O-acetyl-L-serine (L-OAS) from L-serine and acetyl-CoA^9^ (Fig. 1). We sought to test if functional DcsE could be expressed in A549 cells, a model of human type II pulmonary cells^10^. We transfected DcsE tagged with FLAG and Green Fluorescent Protein (GFP) in A549 cells and observed high-level production of DcsE-FLAG-GFP using fluorescence microscopy. We observed in vitro synthesis of L-OAS specifically by purified DcsE using HPLC–MS. We provide evidence that functional DcsE can be synthesized in human cells as a first step for prophylactic D-CS synthesis.

## Results

### D-CS is the optimal candidate for genetic chemoprophylaxis of TB

To identify the optimal antibiotic for synthesis in human cells, we selected for known TB antibiotics with low resistance profiles, short biosynthetic pathways, and precursors present in human cells. We limited our search to the twelve first- and second-line anti-tubercular drugs, due to their known effectiveness against TB (Table 1). We excluded chemotherapeutics and semi-synthetic antibiotics that could not be produced biosynthetically (Table 1). For instance, isoniazid is produced by organic synthesis and there is no known biosynthetic route for production^11^. Likewise, the semisynthetic compound, amikacin is derived from kanamycin A via an acylation reaction with L-(-)-4-amino-2-hydroxybutyric acid which is not detected in the human metabolome^12^. Therefore, because amikacin cannot be produced biosynthetically without additional synthesis of L-(-)-4-amino-2-hydroxybutyric acid, it is excluded. Of the twelve first- and second-line therapies, only capreomycin IA/IB, D-CS, kanamycin A, streptomycin, and Rifamycin SV could be produced in human cells because their precursors were present in human cells and their pathways are fully biosynthetic. Between those pathways, D-CS is the shortest pathway requiring only six steps for synthesis (Table 1). In contrast, capreomycin IA/IB requires fourteen steps, kanamycin requires eight steps, streptomycin requires twenty-five steps, and Rifamycin SV requires twenty-seven steps. D-CS is a second-line antibiotic capable of treating multidrug-resistant TB and is produced by several *Streptomyces* species^13^. *S. lavendulae* produces D-CS using L-serine, acetyl-CoA, L-arginine, and six enzymes encoded by *DcsABCDEG* (Fig. 1). This biosynthetic pathway has been heterologously reproduced in *Escherichia coli* and was shown to synthesize micromolar concentrations of D-CS^14^. Additionally, D-CS inhibits two essential enzymes for bacterial cell wall synthesis, alanine racemase and D-alanyl-D-alanine ligase^15^, which contributes to the low-resistance incidence to D-CS^16^. Because D-CS is synthesized in a relatively short pathway, the precursors are present in human cells, and there is a low resistance incidence ^16^, the D-CS pathway is optimal for the proposed genetic chemoprophylaxis.

**Table 1:** Comparison of antitubercular antibiotics to identify shortest biosynthetic pathways with biologically available precursors.

Drug name	1st or 2nd Line^17^	Antibiotic/ Chemo-therapeutic	MIC for Mtb (µg/mL)	# of Steps for synthesis	# of Precursors*	Mechanism of resistance
Isoniazid	1st	Chemo-therapeutic^11^	0.02–0.2^18–21^	1^11^	2^11^	Inhibitory mutations of peroxidase genes (katG, ahpC)^22,23^, mutations of the promoter region of a key fatty acid synthesis enzyme gene (inhA)^22,24^, and facilitatory mutations in the NADH dehydrogenase gene (ndh)^24,25^
**Rifamycin SV**^**†**^	**1st**	**Antibiotic**^11^	**Rifampicin: 0.05–0.53**^8–21,26–46^ **Rifapentine: 0.015–0.6** ^20,21,21,45^	**27**^47^	**7**^47,48^	**Rifampicin: Mutations that change the binding affinity of the b subunit of bacterial RNA polymerase for rifampicin (rpoB)**^49–51^ **Rifapentine: Mutations of rpoB, often the same as those that confer rifampicin resistance**^49,51^
Pyrazinamide	1st	Chemo-therapeutic^11^	12.5–20^20,52,53^	5^11^	6^11^	Inhibitory mutations of the pyrazinamidase gene (pncA)^54,55^ and mutations that change the binding affinities of ribosomal protein S1 (rpsA)^54,56^ and aspartate l-decarboxylase (panD) for pyrazinamide^54,57^
Ethambutol	1st	Chemo-therapeutic^11^	0.5–2.0 ^19–21,58–60^	3^11^	5^11^	Facilitatory mutations in a gene cluster critical for mycobacterial cell wall synthesis (embCAB)^61,62^ and those in a gene that encodes a protein (DPA) that competitively binds to ethambutol (ubiA)^63,64^
**Cycloserine**	**2nd**	**Antibiotic**^11^	**10–50**^21,60,65^	**6**^14^	**2**^14^	**Mutations causing the overexpression of alanine racemase (alr)**^16,66,67^** and loss-of-function mutations of the L-alanine dehydrogenase gene (ald)**^68^
Ethionamide	2nd	Chemo-therapeutic^11^	0.25–1.25 ^20,21,60^	9^11^	13^11^	Facilitatory mutations in the NADH dehydrogenase gene (ndh)^69^, mutations of the promoter region of a key fatty acid synthesis gene (inhA)^70–72^, and inhibitory mutations of a monooxygenase necessary for ethionamide activation (ethA)^72^
**Streptomycin**	**2nd**	**Antibiotic**^11^	**1.0**^20,21,60^	**25**^73^	**7**^73^	**Mutations to genes that dictate the shape of ribosomal protein S12 (rspL) or 16 s ribosomal RNA (rrs)**^74–76^** as well as inhibitory mutations of a 16S ribosomal RNA methyl transferase (gidB)**^77,78^
Amikacin	2nd	Semi-synthetic Antibiotic^79^	0.5–2.2^20,21,80^	17^79,81^	17^79,81^	Mutations to the genes that dictate the shape of 16S ribosomal RNA (rrs)^82–84^ and loss of function mutations on the gene for a ribosome methyltransferase (tlyA)^85^
**Kanamycin A**	**2nd**	**Antibiotic**^11^	**1.0–5.0 **^21,60,65,86^	**8**^87^	**5**^87^	**Mutations to the genes that dictate the shape of 16S ribosomal RNA (rrs)**^82–84^** and mutations in the promoter region of the gene for an N-acetyltransferase that plays a role in cell survival (eis)**^88,89^
**Capreomycin IA/IB**	**2nd**	**Antibiotic**^11^	**1.0–7.0**^20,21,90^	**14**^91^	**7**^91^	**Mutations to the genes that dictate the shape of 16S ribosomal RNA (rrs)**^84,85^** and loss of function mutations on the gene for a ribosome methyltransferase (tlyA)**^92,93^
Para-amino salicylic acid	2nd	Chemo-therapeutic^11^	0.45–2.0 ^20,60,94,95^	1^11^	3	Loss of function mutations in the gene for a thymidylate synthase critical to thymine biosynthesis and the folate pathway (thyA)^96–98^
Levofloxacin	2nd	Chemo-therapeutic^99^	0.5^20,100,101^	8^99,102^	8^99^	Mutations in the DNA gyrase subunit A gene that reduces levofloxacin binding affinity (gyrA)^103,104^

Pathways with biologically available precursors in bold.

* Commonly available small molecules, such as, H_2_, O_2_, and H_2_O were not counted as antibiotic precursors. Common cofactors such as NAD^+^, ATP, FAD, and acetyl CoA were also not listed as antibiotic precursors.

^†^ Rifamycin SV has several derivatives. These include, notably, Rifampicin, Rifapentine, and Rifabutin^105^.

### A549 cells express soluble and insoluble DcsE-FLAG-GFP

**Figure 2:**
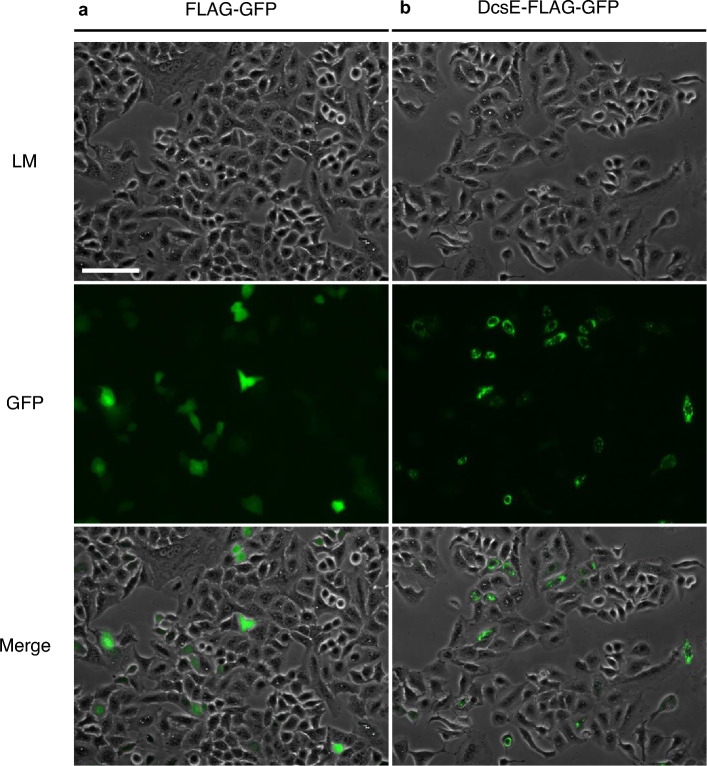
A549 cells express FLAG-GFP and DcsE tagged with FLAG and GFP. (**a**) GFP in FLAG-GFP transfected A549 cells (negative control) indicates expression of GFP-FLAG and more diffused protein. (**b**) GFP in DcsE-FLAG-GFP transfected A549 cells indicates more punctate expression of DcsE-FLAG-GFP. Light microscopy (LM), Green Fluorescent Protein (GFP). Scale bar is 100 µm.

We sought to test if DcsE, the first enzyme of the *S. lavendulae* D-CS biosynthetic pathway, is functional when synthesized in human cells. To express DcsE-FLAG-GFP in human lung (A549) cells, we transfected the cells with plasmid DNA containing DcsE-FLAG-GFP or FLAG-GFP as a negative control. GFP fluorescence can be used as a measure of whether DcsE is expressed and its location within the cell. Following twelve hours of transfection, we observed diffuse GFP fluorescence in the FLAG-GFP transfected cells (Fig. 2a). In contrast, we observed both diffuse GFP fluorescence as well as puncta in the DcsE-FLAG-GFP transfected cells (Fig. 2b). Based on these observations, we conclude DcsE-FLAG-GFP expresses in A549 cells.

### Detection of L-OAS using HPLC–MS/MS

**Figure 3:**
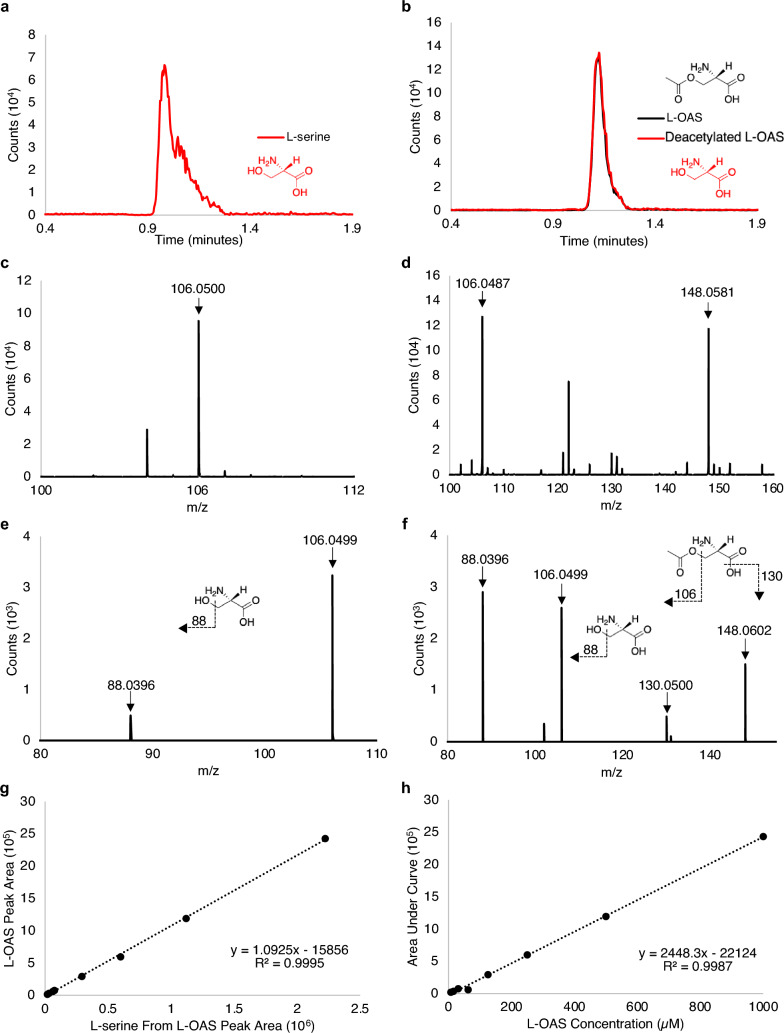
Retention times, MS, MSMS results, and standard curves for L-serine and L-OAS in reaction buffer using HPLC–MS. (**a**) HPLC chromatogram showing retention time vs counts for 500 µM L-serine (0.9 min). (**b**) HPLC chromatogram of a pure 125 µM L-OAS standard. L-OAS (black) and the fragmentation of L-OAS to L-serine (red) appear at the retention time of 1.1 min. (**c**) ESI-TOF positive mode peak for L-serine 106.05 m/z (molecular weight (MW) of L-serine 105.09). (**d**) ESI-TOF positive mode peak for L-OAS 148.0581 m/z (MW of L-OAS 147.13) and fragmented L-OAS to L-serine 106.0499 m/z. (**e**) Tandem MS for L-serine at 5 V shows a single fragment of 88.0396 m/z and intact L-serine at 106.0499 m/z. (**f**) Tandem MS for L-OAS at 5 V show fragments of 88.0396, 106.0499, 130.0500, and intact L-OAS at 148.0602 m/z. (**g**) Linear relationship between peak area of L-OAS from standard curve and the peak area of fragmented L-OAS to L-serine. (**h**) Standard curve of L-OAS (concentrations ranging from 1 mM to 7.8 µM). Inset chemical schematic depict fragmentation consistent with fragment ions observed.

We developed a method to detect L-OAS and the reactants in a reaction buffer suitable for DcsE using HPLC–MS/MS. We observed specific retention times for L-serine at 0.9 min (Fig. 3a), L-OAS at 1.1 min (Fig. 3b), and acetyl CoA (Fig. S1) with masses that correspond to L-serine and L-OAS (Figs. 3c–f). A parent ion of an L-serine standard yielded a mass of 106.049 m/z and fragments into a mass of 88.0396 m/z which is consistent with the loss of an alcohol group (Fig. 3e). A parent ion of an L-OAS standard yielded a mass of 148.06 m/z with three fragment ions of 130.05 m/z (consistent with the loss of an alcohol group from L-OAS), 106.049 m/z (consistent with the loss of the acetyl group on L-OAS), and 88.0396 m/z (consistent with the loss of an alcohol group from deacetylated serine; Fig. 3f).

A pure L-serine standard reveals only a mass for L-serine at 0.9 min (Fig. 3c). A pure L-OAS standard reveals masses of both L-serine and L-OAS at the retention time of 1.1 min with no serine peak at 0.9 min (Fig. 3b,d). This observation is consistent with deacetylation of the L-OAS standard into L-serine by the electrospray ionizer following HPLC elution. If serine were present in the original L-OAS standard, a separate retention time would have been observed.

To test if de-acetylation by ESI affects the quantification of L-OAS, we compared the ratio of L-serine peak area (at 1.1 min) and L-OAS peak area (at 1.1 min) across our L-OAS standard curve. We observed that the fragmentation of L-OAS into L-serine is proportional to concentration (Fig. 3g). Therefore, our method is suitable for L-OAS detection and quantification (Fig. 3h).

### Heterologous DcsE-FLAG-GFP catalyzes reaction of L-serine and acetyl-CoA to O-acetyl-L-serine in Vitro

**Figure 4:**
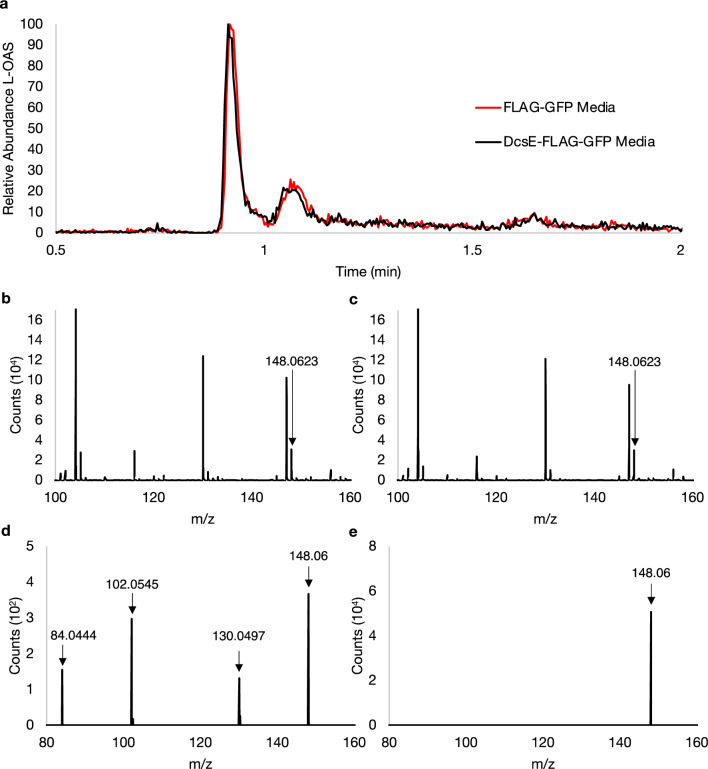
No detection of L-OAS from the media of A549 cells transfected with DcsE-FLAG-GFP or FLAG-GFP. (**a**) Chromatogram of suspected L-OAS (148 m/z) detected from FLAG-GFP (red) and DcsE-FLAG-GFP (black) transfection media. (**b**, **c**) ESI-TOF peaks of 148.058 m/z representing suspected L-OAS at 1.14 min from FLAG-GFP (b) and DcsE-FLAG-GFP (**c**) transfection media. (**d**,**e**) Tandem MS of 148 m/z from (b) and (c), respectively, show fragments of 84.0444, 102.0545, 130.0497, and 148.06 m/z at 5 V. The expected 88.0396 and 106.05 fragment ions from an L-OAS standard were not detected.

After observing expression of DcsE-FLAG-GFP in A549 cells, we sought to determine if L-OAS could be detected from the transfection media. Media from A549 cells expressing DcsE-FLAG-GFP and FLAG-GFP as a negative control was analyzed using HPLC–MS to detect L-OAS. L-OAS was not detected from the media of cells transfected with DcsE-FLAG-GFP compared to FLAG-GFP (Fig. 4). In both samples, a mass of 148.058 m/z was detected with similar relative abundance (Fig. 4b,c), however, insufficient fragment ions associated with an L-OAS standard (Fig. 3f) were identified (Fig. 4d,e). A fragment of 130 m/z was detected from the FLAG-GFP transfection media indicative of a loss of an alcohol group from the compound with a mass of 148 m/z; however, the serine peak (106 m/z) and the serine fragment ion were not detected (Fig. 4d). No fragment ions were detected from DcsE-FLAG-GFP transfection media indicating that the compound with a mass of 148 m/z was not L-OAS (Fig. 4e). Taken together, we conclude L-OAS could not be detected from the transfection media.

**Figure 5:**
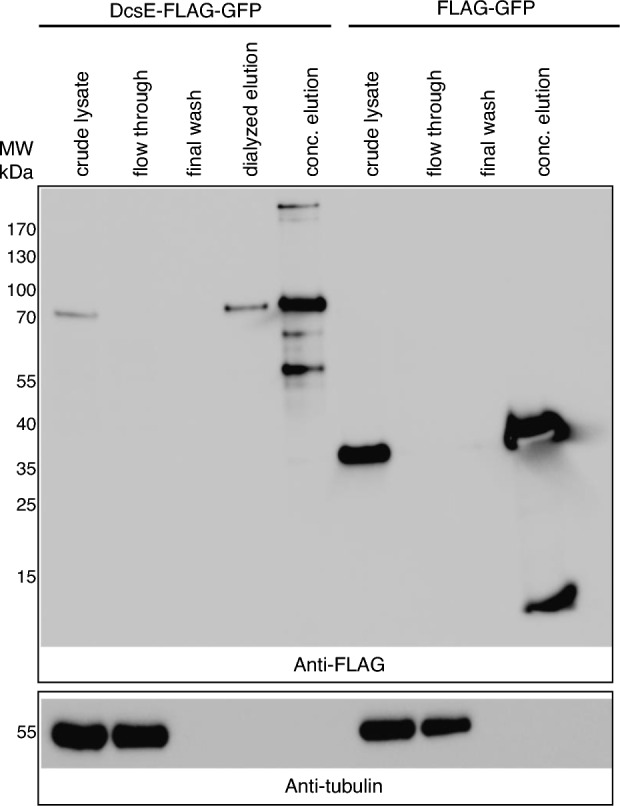
DcsE-FLAG-GFP is purified and concentrated following FLAG immunoprecipitation. Western blot analysis of indicated fractions from purification of DcsE-FLAG-GFP (67 kDa) or FLAG-GFP (28 kDa) from transfected A549 cells. 7 µl of indicated fraction was loaded per lane. Membrane was first probed with anti-FLAG antibody and then stripped and reprobed using anti-tubulin as detailed in materials and methods. Full sized blots and ladder images are provided as supplementary data (Fig. S2).

To understand if DcsE-FLAG-GFP produced in A549 cells is capable of catalyzing the reaction of L-serine and acetyl-CoA to form L-OAS, we purified the enzyme from cells using the FLAG tag. To determine if FLAG-GFP and DcsE-FLAG-GFP were purified following immunoprecipitation, we performed immunoblot analysis on whole cell lysate and select fractions from immunoprecipitation (see materials and methods and Fig. 5). As expected, DcsE-FLAG-GFP (67 kDa) and FLAG-GFP (28 kDa) are observed in their respective lysates (Fig. 5). FLAG-GFP and DcsE-FLAG-GFP are not detected in the flow through or washes indicating FLAG affinity gel captured expressed protein. Following elution with 3 × FLAG peptide, presence of FLAG-GFP and DcsE-FLAG-GFP indicate 3 × FLAG peptide elutes the targeted protein. The presence of extra bands in the concentrated elutions indicates partial protein degradation. The presence of tubulin in lysate and flow through fractions and lack of tubulin in elutions indicates FLAG-GFP and DcsE-FLAG-GFP are purified from other cellular proteins (Fig. 5). Together these results indicate FLAG precipitation and elution purifies FLAG-GFP and DcsE-FLAG-GFP.

**Figure 6:**
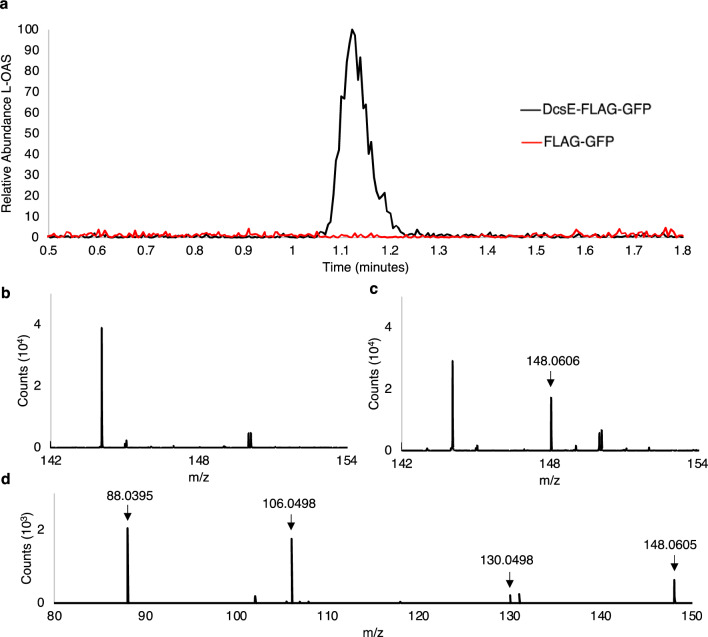
Synthesis of D-CS intermediate, L-OAS, by heterologous purified DcsE-FLAG-GFP. Purified DcsE reacted with L-Serine and Acetyl-CoA was functional as determined by presence of enzyme product, L-OAS using HPLC–MS as analyzed by MassHunter**.** (**a**) HPLC chromatogram of L-OAS produced by DcsE-FLAG-GFP (in black) at 1.117 min. L-OAS was not detected from a reaction containing FLAG-GFP (in red). (**b**) FLAG-GFP containing samples produce no detectable peak of 148.06 m/z at 1.117 min suggesting no L-OAS production. (**c**) DcsE-FLAG-GFP containing samples produce ESI-TOF peak of 148.06 m/z at 1.117 min which corresponds to mass of L-OAS. (**d**) tandem MS of L-OAS produced by DcsE create fragments at 88.04, 106.05, and 131.03 m/z.

To test if purified DcsE produced in A549 cells can catalyze the reaction of L-serine and acetyl-CoA to L-OAS, we reacted L-serine and acetyl-CoA in the presence of purified DcsE-FLAG-GFP or FLAG-GFP as a control and used HPLC–MS to detect the expected product L-OAS. We observed L-OAS in the sample containing DcsE-FLAG-GFP but not FLAG-GFP (Fig. 6a). L-OAS produced by DcsE-FLAG-GFP had a retention time of 1.117 min (Fig. 6a) and 148.0606 m/z (Fig. 6c). Tandem MS shows L-OAS produced by DcsE-FLAG-GFP fragments into 131.0335, 106.0498, and 88.0395 m/z fragments (Fig. 6d), consistent with the tandem MS data for an L-OAS standard (Fig. 3f). No L-OAS was detected in the FLAG-GFP reaction (Fig. 6b). Simultaneously, we ran a standard curve of L-OAS (Fig. 3h) to calculate the L-OAS produced by DcsE-FLAG-GFP. We observed DcsE-FLAG-GFP produced 40.3 µM L-OAS after a 1 h reaction at 30 °C. From this we conclude active heterologous production of DcsE-FLAG-GFP in A549 cells.

## Discussion

In this study, we observed that human lung cells transfected with DcsE-FLAG-GFP are capable of synthesizing functional DcsE, which catalyzes the first step towards D-CS synthesis. D-CS is an optimal candidate for a genetic chemoprophylaxis against TB because it is the shortest known pathway for an anti-TB antibiotic with biologically available precursors (Table 1). DcsE tagged with FLAG-GFP can be expressed in A549 cells (Fig. 2), but there is no appreciable difference between the L-OAS detected from DcsE-FLAG-GFP and FLAG-GFP transfection media (Fig. 4). However, purified DcsE-FLAG-GFP (Fig. 5) catalyzes the formation of L-OAS from L-serine and acetyl-CoA (Fig. 6). L-OAS produced by purified DcsE-FLAG-GFP has the expected retention time of 1.1 min (Fig. 6a), a mass of 148.13 m/z (Fig. 6c) and produced the same 106 m/z and 131 m/z fragments at 5 V (Fig. 6d) as the 0.5 mM L-OAS standard in reaction buffer (Fig. 3f). No L-OAS was detected from a reaction containing purified FLAG-GFP (Fig. 6b).

Production of an active DcsE enzyme in human lung cells demonstrates progress towards a genetic chemoprophylaxis against TB. Previous studies have shown that other TB antibiotics such as isoniazid can be used prophylactically to prevent TB infection in those infected with HIV and people who have tested positive for TB by Tuberculosis skin test^26^. Isoniazid is not the only TB antibiotic used chemoprophylactically; rifampicin, pyridoxine, and combinations of rifampicin and pyridoxine have also been shown to reduce the incidence of TB infection compared to a placebo^27^.

One limitation to using D-CS for the proposed gene therapy is the comparatively high minimal inhibitory concentration (MIC) for Mtb of 10–50 µg/mL which could restrict the efficacy of the therapy depending on how much D-CS can be produced in humans. In this study, 40.3 µM L-OAS was produced after a 1 h reaction at 30 °C which equates to 5.94 µg/mL L-OAS, meaning the concentration of a precursor is lower than the MIC of D-CS. Furthermore, no L-OAS was detected in media from FLAG-GFP and DcsE-FLAG-GFP transfected cells (Fig. 4), so DcsE and other D-CS biosynthetic genes needs to be optimized.

However, D-CS expression does not need to reach the full MIC concentration to be effective. Typically, D-CS does not reach MIC concentrations in patients, suggesting reaching MIC is not required for therapeutic efficacy. D-CS is known for its poor lung cavity penetration; the clinical dose for adults of 250 mg only reaches concentrations of less than 2 µg/mL in the lung^28^ meaning that small amounts of D-CS being produced intracellularly while infection is mild could be clinically relevant. Future experiments in conditions that more closely model the complex environment of the human system could better determine realistic concentrations of D-CS that could be produced. For example, noncancerous cell models such as Beas-2B, macrophages, and in vivo models could be used. Additionally, in vivo models will better portray how local D-CS expression could protect at the organ level in body systems.

**Figure 7:**
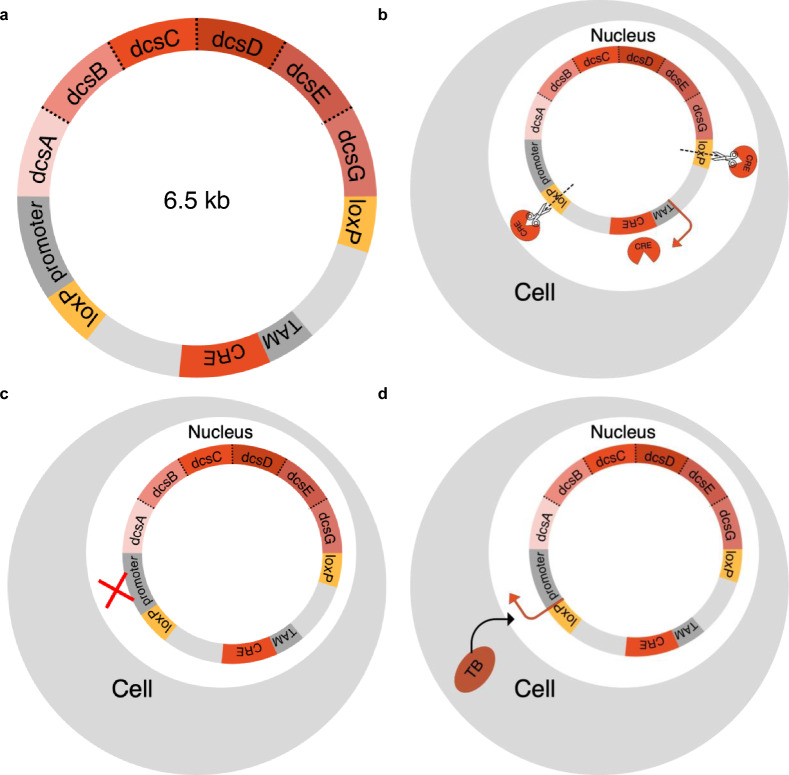
Proposed excisable D-CS plasmid for TB-inducible expression. (**a**) Map of proposed D-CS plasmid. Infection responsive element promoter (grey: *InfRE*), tamoxifen inducible promoter (grey: *TAM*), *DcsABCDEG* and *CRE* genes *(reds)*, excisable *loxP* sites (gold), *Mtb* MycP1 cleavage sites SLKPASAGGG (dotted lines). (**b**) Cell with excised plasmid activated by tamoxifen which expresses CRE recombinase to cut at *loxP* sites. (**c**) Cell not expressing *DcsABCDEG* in the case of no TB infection. (**d**) Cell expressing *DcsABCDEG* in the case of TB infection.

To safely apply genetic chemoprophylaxis for TB, we propose developing an excisable gene therapy that delivers D-CS specifically in the presence of TB infection as depicted in Fig. 7. Such a gene therapy will need to be excisable in the case of adverse effects, inducible in the case of infection, and active specifically in response to TB. The site-specific recombination of the CRE/*loxP1* system could be used to permanently excise *dcsABCDEG* in the case of adverse effects. As visualized in Fig. 7a, expression of the recombinase CRE is activated by the drug tamoxifen^29^. When expressed, CRE cleaves the two *loxP* sites thus excising the construct responsible for production of D-CS (Fig. 7b). The CRE/*loxP1* system is a suitable system for this application because it is precise, controlled by a pharmaceutical that is not naturally present in human cells, and is able to stop D-CS expression quickly and permanently. Additionally, *dcsABCDEG* will be selectively expressed in the presence of infection through an appropriate infection responsive promoter element (*InfRE;* Fig. 7c). For instance, the *MIP-2* promoter could be used to activate the plasmid in the presence of intracellular bacteria such as *Mtb*^30,31^. Because the *MIP-2* promoter is responsive to all intracellular infections, additional molecular engineering will be necessary. To accomplish this, the MycP1 protease^32^, produced by *Mtb,* could be used to control the expression of functional enzymes. By tethering DcsABCDEG with the MycP1 specific polypeptide SLKPASAGGG, we expect active enzyme will only be produced in the presence of TB. By including an infection induced promoter, MycP1 sites between each enzyme to control functionality of enzymes in the presence of TB, and an excisable plasmid controlled by CRE/*loxP1* in our design, the expression of D-CS synthesis genes is controlled.

Another important safety factor is tolerance by the host. As depicted in Fig. 1, DcsA and B synthesize hydroxyurea from L-arginine^14^. Hydroxyurea is known to be cytotoxic to human cells between concentrations of 2–10 mM ^33^. While these concentrations far exceed expected in vivo concentration of D-CS of approximately 50 µM, an important future area of study will be to determine if hydroxyurea reaches toxic levels, or is consumed quickly enough by DcsD to avoid any cytotoxic effects.

The system used to deliver the genetic chemoprophylaxis must be long lasting and well tolerated. Adeno-associated viruses (AAV) are one way to deliver a construct similar to the one proposed in Fig. 7a. AAV are a versatile and effective way of delivering genetic material via a protein shell containing single stranded DNA^34^. The genome capacity of AAV is generally considered to be around 4.5-5 kb which is below the estimated size of the plasmid proposed in Fig. 7. However, AAV5, a serotype of AAV, has been shown to incorporate genome sizes up to 8.9kb^35^ which is larger than the plasmid proposed in Fig. 7. Cationic liposomes (CLs) are another example of a technology capable of delivering a gene therapy. CLs are non-viral and use a closed lipid bilayer membrane to deliver genes and protect the DNA from degredation^36^. CLs are very customizable and can transfer up to 1,000kb^36^ into cells making them another promising technology for gene therapy applications. Overall, both AAV and CLs are delivery methods that could be used to deliver the construct tissue specifically, safely, and dependably. In vivo delivery of gene therapy is an active area of research to increase the relatively low transfection efficiency using AAV and CL^37^. Additionally, future studies would be necessary to determine if macrophages or lung cells would be a better ultimate target for this approach based on the nature of TB infection and the transfection efficiency of each cell type.

By observing heterologous production of functional DcsE-FLAG-GFP, we provide evidence that the first step for prophylactic D-CS synthesis is possible in human cells. Our immediate future directions include developing a method to observe this enzymatic activity in live A549 cells and eventually advance to synthesizing all six enzymes in the D-CS pathway via the proposed plasmid. Additionally, future studies could replicate this study in noncancerous cell models such as Beas-2B or macrophages. Finally, in vivo models would ultimately be necessary to determine the efficacy of the proposed genetic chemoprophylaxis. Overall, with the rapid growth of cell-based therapies, we seek to create the tools now to prepare for a future state where gene therapies are commonplace, safe, economically plausible, and well accepted.

## Materials and methods

### Cell lines and cell culture

A549 respiratory epithelial cells (ATCC CCL-185) were maintained in complete F-12 K Medium comprised of Kaighn's Modification of Ham's F-12 K Medium (ATCC 30–2004), 10% FBS (v/v; VWR 89,510–186), and 1% penicillin–streptomycin (v/v; ATCC 30–2300). Cells were maintained according to ATCC handling guidelines for A549 cells^38^.

### Cloning GFP-FLAG-DCSE

The DcsE gene is present in the D-cycloserine synthesizing bacterium *Streptomyces lavendulae. S. lavendulae* (ATCC #11,924) was obtained from ATCC in freeze dried form, and was reanimated according to supplier instructions (ATCC, n.d.-c). The organism was revived in yeast malt extract broth (5 g dextrose, 2.5 g peptone, 1.5 g malt extract, and 1.5 g yeast extract made up to 500 ml in diH2O) and streaked on the same agar media, with growth occurring two days after inoculation at 26 °C.

A fresh liquid culture of *S. lavendulae* was then cultured from a single colony, and following growth, the cells were lysed. 5 ml of cells were pelleted and washed in 200 µl of lysozyme digestion buffer. Then, the cells were transferred to a screw cap centrifuge tube containing 0.1 mm glass beads and homogenized for 30 s. Genomic DNA was then extracted from the bacteria using the PureLink Genomic DNA Minikit (catalog #k182001). The final eluted solution was analyzed using a nanodrop and revealed the clean peak at 260 nm characteristic of DNA. Two concentration readings were performed yielding an approximate concentration of 15 ng/ µl.

DcsE was first cloned into the vector pCOOFY50 (Addgene plasmid #55,189) by Gibson assembly (https://www.nature.com/articles/nmeth.1318 ) using the NEB HiFi DNA system (catalog # E2621S). 20 ng of PCOOFY50 backbone was amplified using the primers AB0001 and AB0002 (Table 2) and pHusion high-fidelity polymerase (NEB # M0530S) according to the PCR protocol described in Table 3. Likewise, a DcsE fragment was amplified using 50 ng of genomic DNA from *S. lavendulae* and primers AB0009 and AB0010 using the same protocol. After PCR product cleanup using the Zymo clean and concentrator kit (Zymo #D4004), homologous ends of the two products were annealed using the NEB HiFi DNA system (catalog # E2621S) and transformed into DH5α *E. coli* as described below.

**Table 2:** Primers used for this study.

Primer name	Target	Use	Sequence
AB0001	pCOOFY50 backbone	RP for pCoofy50	GGGCCCCTGGAACAGAACTTCCA
AB0002	pCOOFY50 backbone	FP for pCoofy50	CGCCATTAACCTGATGTTCTGGGG
AB0009	DcsE from *S. lavendulae*	FP for DcsE	aagttctgttccaggggcccAGGGAATTCATACCCCCGG
AB0010	DcsE from *S. lavendulae*	RP for DcsE	agaacatcaggttaatggcGTCAGGCGACGATCGCCA
ASB001	PCS2 + FLAG-GFP backbone	FP for pCS2 + backbone	AGGCGCGCCGATTATAAAG
ASB002	PCS2 + FLAG-GFP backbone	RP for pCS2 + backbone	CATGGTGGCGAATTCGAATC
ASB011	DcsE from pCOOFY50 vector	FP for DcsE	gattcgaattcgccaccatgAGGGAATTCATACCCCCGG
ASB012	DcsE from pCOOFY50 vector	RP for DcsE	tctttataatcggcgcgcctGGCGACGATCGCCAGGAA

**Table 3:** Cycling conditions for gradient PCR. Reactions were run as needed, and ranges of annealing temperature were tested (see table 3 for annealing temperatures used).

#	Purpose	°C	sec
1	Initial Denaturing	98	30
2	Denaturing	98	10
3	Annealing	69	15
4	Extension	72	Variable
5	Return to step 2 30 times		
6	Final extension	72	600

Neither DcsE nor GFP expressed using the pCOOFY50 vector, so DcsE was subcloned into pCS2 + (https://www.addgene.org/vector-database/2295/ ) containing FLAG-GFP downstream of the CMV promoter. DcsE was subcloned into pCS2 + using PCR amplification followed by Gibson assembly as described above with the following modifications: Primers ASB001 and ASB002 were used to amplify pCS2-FLAG-GFP backbone and primers ASB011 and ASB012 were used to amplify DcsE from pCOOFY50 DcsE. The resultant plasmids were named pCS2-FLAG-GFP and pCS2-DcsE-FLAG-GFP.

The promoter sequences and open reading frames of plasmids were verified by Sanger sequencing at the University of Chicago Comprehensive Cancer Center (UCCC) sequencing facility.

## Plasmid purification for transfection

To transform competent DH5α with DcsE-FLAG-GFP or FLAG-GFP, 50 µl of competent DH5α and 2 µl of purified plasmid were combined and incubated on ice for 30 min. Samples were heat shocked at 42 °C for 45 s and placed on ice for 5 min. 800 µl SOC was added and shaken for one hour at room temperature. 150 µl of this solution was plated on LB agar plates with 100 µg/ml ampicillin and grown overnight at 37 °C. One colony was transferred to 5 mL sterile LB broth with 100 µg/ml ampicillin and shaken at 37 °C for 12 h. The ZymoPURE Plasmid Miniprep Kit was used following the centrifugation protocol to purify pure plasmid.

### Transfection of DcsE-FLAG-GFP

To transfect A549 cells with DcsE-FLAG-GFP or FLAG-GFP, cells were plated on tissue culture treated 60 mm plates at a concentration of 176,800 cells/mL in complete F-12 K Medium. 12 h after plating, cells were transfected using Lipofectamine 3000 (Thermo Fisher L3000) and 6ug purified plasmid following the Thermo Fisher guidelines for A549 cells^39^. Cells were transfected overnight for 12 h at 37 °C at 4.5% CO_2_. Following 12 h of transfection, media was collected saved for subsequent analysis.

### Imaging transfected cells

A549 cells were imaged 12 h following transfection using a Nikon Eclipse TE2000-U inverted phase contrast microscope (Tokyo, Japan) with a Nikon TE2-PS100W power supply, a Chiu Technical Corporation 100-W mercury lamp (Kings Park, USA), and Nikon NIS-Elements software. Cell images were taken using the 10X objective, the 1.5X bonus zoom, and phase 1. For each position, an image was taken using differential interference contrast (DIC) to focus the cells and blue light to image GFP fluorescence. ImageJ was used to merge images.

### Preparation of lysate

A549 cells were lysed immediately following transfection and imaging. Media was removed and cells were washed with 5 mL PBS (ATCC 30–2200). 150 µl cold radioimmunoprecipitation assay buffer (RIPA) lysis buffer containing 150 mM NaCl, 1% IGEPAL CA-630 (NP40), 0.5% sodium deoxycholate, 0.1% SDS (sodium dodecyl sulphate), 50 mM Tris–HCl, pH 8.0 was added to each plate. Cells were lysed on ice for about 30 s using a cell scraper. An inverted microscope was used to ensure all cells were lysed. All the liquid was transferred to prechilled microcentrifuge tubes and centrifuged at 13,000xg for 10 min at 4 °C. The supernatant was transferred to a new tube and purification immediately followed.

### DcsE-FLAG-GFP lysis and purification

Cell lysate was immediately purified to remove proteases in crude lysate. Protein was purified using Sigma Monoclonal ANTI-FLAG M2 Affinity Gel and the sigma protocol “Immunoprecipitation of FLAG Fusion Proteins Using Monoclonal Antibody Affinity Gels” was followed. TBS (0.5 M Tris HCl, pH 7.5, 1 M NaCl) was used to wash gel prior to immunoprecipitation. Samples were eluted three times using 3 × FLAG peptide (Rockland Immunochemicals) at a working concentration of 150 ng/µL in TBS. After elution, samples were filtered using 500 µl 10 kDa microcentrifuge filter (Amicon) to filter out excess 3 × FLAG peptide, exchange lysis buffer to 10 × reaction buffer containing 40 mM Tris–HCl (pH 7.6), 400 mM NaCl, 2 mM EDTA, modified from and to concentrate samples to a final volume of 50 µl.

### Immunoblotting

Presence of FLAG-GFP and DcsE-FLAG-GFP were detected using the abcam Western blot protocol^40^. 7 µl of each fraction was loaded per lane along with 2.5 µl 4 × LDS sample buffer (Thermo Fisher NP0007). 5 µl PageRuler prestained protein ladder was used as a reference (Thermo Fisher 26,616) A NuPAGE 4–12% Bis–Tris Gel (Thermo Fisher NP0321BOX) was used with NuPAGE MES SDS running buffer (Thermo Fisher NP0002) according to the manufactures instructions^41^. Gels were transferred to a PVDF membrane using a Bio-Rad Trans-Blot SD Semi-Dry Transfer Cell at 25 V for 30 min with Bjerrum Schafer-Nielsen Transfer buffer: Tris Base 48 mM and Glycine 39 mM (pH 9.2). The membrane was blocked in 3% BSA (w/v) in PBS 0.1% Tween 20. Primary anti-FLAG (Sigma F1804) was diluted 1:1000 in 3% BSA and the secondary anti-Mouse (Thermo Fisher 31,431) was diluted 1:10,000. ECL substrate (BioRad 1,705,062) was used according to manufacturer’s instructions^42^ with a BioRad ChemiDoc Imaging System. Western blots were stripped and reprobed using the abcam stripping for reprobing protocol^43^. Primary anti-Tubulin (NOVUS NB600-936) was diluted 1:1000 and secondary anti-Rabbit (Promega W401B) was diluted 1:2000. The blots were imaged as described above.

### In vitro L-OAS synthesis

To perform in vitro L-OAS synthesis^14^, 20 µl of purified DcsE-FLAG-GFP and FLAG-GFP were reacted in a 50 µl reaction at a final concentration of 1 mM L-serine, and 1 mM acetyl-CoA in a reaction buffer^44^ containing 4 mM Tris–HCl (pH 7.6), 40 mM NaCl, 0.2 mM EDTA, and 1 mM dithiothreitol (DTT). Samples were reacted for 1 h at 30 °C.

### HPLC mass spectrometry

HPLC–MS was used to detect L-serine and L-OAS from In vitro L-OAS synthesis samples. Samples were separated using an Agilent 1290 Infinity II HPLC with an YMC ODS-AQ 2.0 mm × 100 mm, 5 μm (YMC America) aqueous C18 column and an Agilent 6545 Quadrupole Time of Flight LC/MS mass spectrometer in positive mode at the Integrated Molecular Structure Education and Research Center (IMSERC) facilities at Northwestern University. 1 µl of samples were injected starting with a concentration of 100% solvent A (H_2_O + 0.1% formic acid (FA)) and 0% solvent B (ACN + 0.1% FA). The ratio of solvent B was linearly increased to 50% from 2.0 to 7.0 min. From 7.0 to 9.0 min the ratio of solvent B was again linearly increased to 99%. From 11.0 min to 11.5 min solvent A was increased from 1.0% to 100%. Solvent A remained at 100% until the total method reached 16 min. The flow rate was set to 0.3 mL/min and all samples were run at room temperature.

This method was used to create a standard curve of 1:1 serial dilutions for L-serine and L-OAS ranging from 500 uM to 3.9 uM. Reacted samples were prepared by adding an equal volume of 0.1% FA (Sigma 5.43804) in acetonitrile (Sigma 34,851) immediately after reaction completion. Samples were vortexed and centrifuged at top speed for 10 min and then 75 µl of the supernatant was transferred to autosampler vials. Using the same method as above, HPLC–MS was used to detect product in samples. For tandem MS, voltages of 2.0 and 5.0 V were used. MassHunter Data Acquisition software was used to operate the instrument and MassHunter Qualitative Analysis was used for data analysis (Agilent MassHunter Quantitative Analysis, Version 10.0, Build 10.0.10305.0. RRID:SCR_016657).

## Supplementary Information

Supplementary Information.

## Data Availability

The underlying data for this work are available from the corresponding author upon reasonable request.
