# Supplementary material for: Heterologous production of the D-cycloserine intermediate O-acetyl-L-serine in a human type II pulmonary cell model
Source: Sci Rep. 2023 May 26;13:8551. doi: 10.1038/s41598-023-35632-4 (PMC10214352; doi:10.1038/s41598-023-35632-4)
Supplement: Supplementary file 1 — Supplementary Information. [file 41598_2023_35632_MOESM1_ESM.docx]

**Supplementary Figure 1**. Full chromatogram of L-serine with a retention time of 0.9 min (red), L-OAS with a retention time of 1.1 min (black), and acetyl-CoA with a retention time of approximately 7 min (blue).


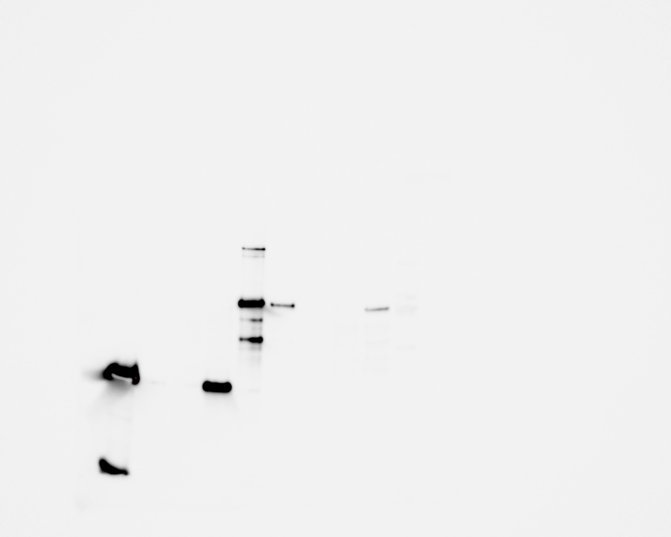

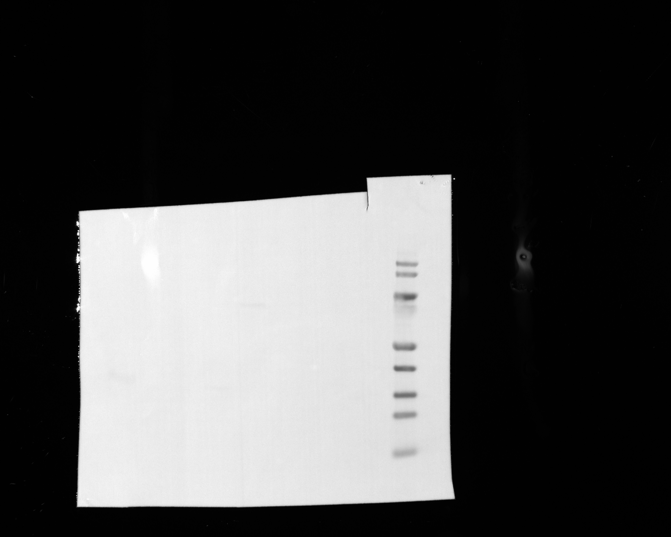

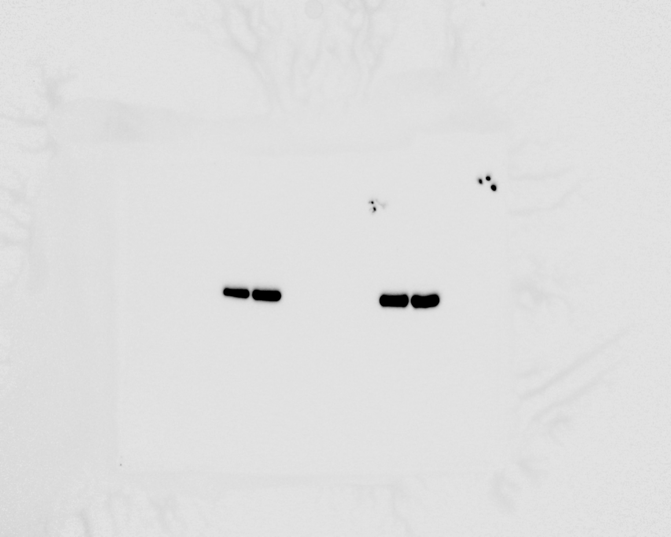

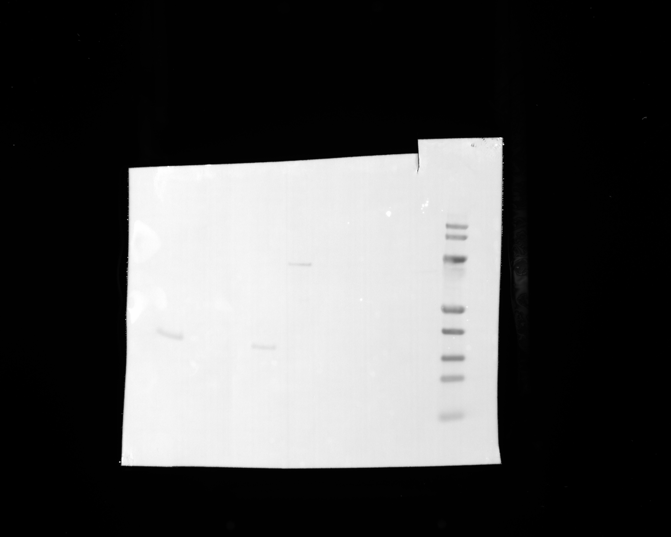


**Supplementary Figure 2.** Full length blot images for figure 3. DcsE-FLAG-GFP fractions were loaded in lanes 1-5. FLAG-GFP fractions were loaded in lanes 6-9 and 5 μl PageRuler Prestained protein ladder (Thermo Fisher 26616) was loaded in lane 10. 7 μl of fractions were loaded as follows: crude lysate (1), flow through (2), final wash (3), dialyzed elution (4), concentrated elution (5), crude lysate (6), flow through (7), final wash (8), and concentrated elution (9). Upper left panel: immunoblot of mouse anti-FLAG antibody. Upper right panel: image of membrane depicting molecular weight ladder. Lower left panel: immunblot of rabbit anti-tubulin antibody. Lower right panel: image of membrane depicting molecular weight ladder. Membrane was stripped and reprobed prior to tubulin immunoblot as describe in materials and methods. Ladder images and immunoblots were overlaid for kDa references in figure 3. Specific antibodies and dilutions are detailed further in materials and methods.
